# Physiological Responses to Firefighting in Extreme Temperatures Do Not Compare to Firefighting in Temperate Conditions

**DOI:** 10.3389/fphys.2017.00619

**Published:** 2017-08-23

**Authors:** Stephanie Windisch, Wolfgang Seiberl, Daniel Hahn, Ansgar Schwirtz

**Affiliations:** ^1^Department of Biomechanics in Sports, Technical University of Munich Munich, Germany; ^2^Human Movement Science, Ruhr-University Bochum Bochum, Germany; ^3^School of Human Movement and Nutrition Sciences, University of Queensland Brisbane, QLD, Australia

**Keywords:** simulated firefighting in extreme temperatures, firefighting performance model, strength and endurance tests, firefighter fitness, aerobic anaerobic metabolism during firefighting

## Abstract

**Purpose:** The aim of this study was to examine physiological responses to two different simulated firefighting exercises: a firefighting exercise with flashovers, smoke, poor visibility and extreme temperatures (300°) in a burning container and a standard firefighting exercise in temperate conditions. Furthermore, a second purpose of the study was to find out if the contribution of strength and endurance capacities to firefighting performance changes when the demands of the firefighting exercise change.

**Methods:** Sixteen professional firefighters performed a maximum treadmill test, strength testing, a standard simulated firefighting exercise (SFE) without heat and flashovers and a firefighting exercise with a simulation of the flashover phenomenon in a burning container (FOT). The treadmill testing was used to determine peak oxygen uptake (VO_2peak_), ventilatory threshold (VT1) and respiratory compensation point (RCP). Three intensity zones were identified according to heart rate (HR) values corresponding to VT1 and RCP: zone 1–HR below VT1, zone 2-HR between VT1 and RCP, zone 3–HR above RCP. Firefighting performance was determined by a simple time-strain-air depletion model (TSA) taking the sum of z-transformed parameters of time to finish the exercise, strain in terms of mean heart rate, and air depletion from the breathing apparatus. Correlations were then established between TSA based firefighting performance parameters and fitness variables representing strength and endurance.

**Results:** HR was significantly lower during SFE (79.9 ± 6.9%HR_max_) compared to FOT (85.4 ± 5.2%HR_max_). During SFE subjects spent 24.6 ± 30.2% of time in zone 1, 65.8 ± 28.1% in zone 2 and 9.7 ± 16.6% in zone 3. During FOT subjects spent 16.3 ± 12.8% in zone 1, 50.4 ± 13.2% in zone 2 and 33.3 ± 16.6% in zone 3. Out of all correlations, relative VO_2peak_ showed the highest relation to mean HR during SFE (−0.593) as well as FOT (−0.693).

**Conclusions:** Endurance in terms of VO_2peak_ is an important prerequisite for both firefighting exercises. However, for standard simulated firefighting exercises it is important to work below VT1. For firefighting exercises in extreme temperatures with smoke, poor visibility and unexpected flashovers a high fitness level is required in order to keep the time spent above RCP as short as possible.

## Introduction

Firefighting is an occupation characterized by sudden bouts of high-intensity workloads when firefighters respond to an emergency. Previous studies revealed that firefighters showed physiological responses of 80% heart rate maximum (HR_max_) on average with a range from 60 to 90% HR_max_ (e.g., von Heimburg et al., 2006; Del Sal et al., 2009; Williams-Bell et al., 2009; Perroni et al., 2010). Researchers found these values while conducting simulated firefighting tasks carried out at a safe and efficient pace. Furthermore, the majority of the reported data were collected without environmental stressors such as extreme temperatures caused by fire. Firefighters are also required to perform sudden exercises in hot and extreme environments accompanied by smoke and poor visibility. Therefore, it is important to know which job-related physiological fitness requirements they need for carrying out firefighting tasks in a safe, healthy and efficient manner. Based on the established job demands for standard workload bouts for the routine job, researchers varied in their recommendations for fitness levels such as a minimum of peak oxygen uptake (VO_2peak_) between 39 and 45 ml/min/kg (O'Connell et al., 1986; Gledhill and Jamnik, 1992; Siddall et al., 2016).

Maintaining the proposed fitness levels means that standard workload bouts can be completed safely. However, as stated by Astrand et al. (2012), the level of fitness should be well above the one required for completing the job routine as researched in simulated firefighting exercises. This extra level of fitness can be required when the exposure to heat represents an additional burden for the cardiovascular system leading to reduced productivity and increased exertion (Larsen et al., 2015). This was observed in some studies reporting more than 88% HR_max_ during simulated firefighting with thermal stress or actual emergencies (Barnard and Duncan, 1975; Sothmann et al., 1992; Smith et al., 1997) while simulated firefighting without heat averaged at 80% HR_max_ (von Heimburg et al., 2006; Del Sal et al., 2009; Perroni et al., 2010). Furthermore, the unpredictability of changing situations in real fire emergency scenes can be an additional stressor. Feared hazards are, for example, suddenly occurring flashovers. This happens when a fire reaches its ignition temperature and spreads rapid unexpectedly.

Firefighters' responses to heat in combination with suddenly occurring dangerous situations such as a flashover have hardly been established yet. A few studies documented significantly higher physiological strain of firefighting exercises in hot environments (up to 100°) compared to those without heat in temperate conditions (<40°) (Smith et al., 1996, 1997; Larsen et al., 2015) but flashover training was not part of these studies. Additionally, they did not provide any information on how endurance and strength variables were related to firefighting in distinct thermal environments.

In a previously published study (Windisch et al., 2017), we investigated a simulated standard firefighting exercise (SFE) without heat, smoke, poor visibility and unpredictable situations such as flashovers and determined firefighting performance by a time-strain-air depletion model (TSA-model). By conducting the present study, we now seek to understand whether and how physiological strain changes when performing a flashover training (FOT) in a burning container including smoke, poor visibility, extreme temperatures and unpredictable situations like flashovers. Both exercises, the SFE and the FOT, are mandatory standard exercises for professional firefighters in Germany and claim to represent firefighting job demands, albeit they are quite different. Therefore, we defined two main objectives for this research: (1) To examine the extent to which the two different simulated firefighting exercises impact on physiological responses of firefighters. (2) To show whether and to what extent the importance of strength and endurance capacities change when the demands of the firefighting exercise change. To define firefighting performance, we used our previously established model, the TSA score (Windisch et al., 2017). Understanding the fitness contribution with respect to the different characteristics of various firefighting exercises enables firefighters to properly prepare for the job requirements.

## Materials and methods

### Study subjects

Sixteen professional firefighters from Munich Airport volunteered for this research (39 ± 9 yr, 176.9 ± 0.1 cm, 82.1 ± 7.6 kg, BMI 26.3 ± 2.4 kg/m^2^). Mean service time of participants was 17 ± 8 years. All participants were in possession of a valid G26.3 medical examination for operational fitness, a mandatory periodically medical health check for professional firefighters in Germany. The G26.3 medical examination includes an eye test, a hearing test, an exercise electrocardiogram, a blood test and a pulmonary function test.

### Experimental design

All participants completed four tests on four different days to investigate differences in physiological responses to firefighting with and without the presence of extreme heat and which fitness parameters were sensitive for both exercises.

#### Treadmill testing

Subjects were dressed in T-shirts, shorts and training shoes. Minute ventilation (V_E_) and gas exchange (oxygen consumption—VO_2_, carbon dioxide output—VCO_2_, respiratory exchange ratio - RER) were measured breath-by-breath with the Cortex Metamax 3B (Cortex Biophysics GmbH, Germany). The incremental exercise test based on the Ellestad Protocol (Ellestad et al., 1969) was conducted on a motorized treadmill (Life Fitness, Integrity Series, Germany) to determine peak oxygen uptake (VO_2peak_), total time to exhaustion and heart rate maximum (HR_max_). The test was terminated when subjects reached volitional fatigue and were not able to continue running. VO_2peak_ and HR_max_ were taken as the highest 30 s-average during the final minute of the test. In addition, based on the test, two thresholds were determined: ventilatory threshold 1 (VT1) and respiratory compensation point (RCP). The VT1 was determined from the V-slope method (Beaver et al., 1986) in combination with the break point of the ventilatory equivalent for O_2_ against VO_2_ (Oshima et al., 1997). The RCP was identified by the break points of the ventilatory equivalent for CO_2_ and the end tidal CO_2_ concentration against VO_2_ (Oshima et al., 1997). VT1 indicates the first turnpoint of ventilation (V_E_) and ventilatory equivalent ratio for oxygen (V_E_/VO_2_) (Wassermann and McIlroy, 1964). In contrast, RCP indicates the maximal lactate steady state, equivalent to the second turn point for V_E_ and V_E_/VO_2_. These two thresholds were then used to establish three physiological intensity zones that correspond to the heart rates at the following exercise intensities: HR below VT1 (Zone 1), HR between VT1 and RCP (Zone 2) and HR above RCP (Zone 3) (Windisch et al., 2017).

#### Standard simulated firefighting exercise (SFE)

This exercise is a standardized, mandatory and periodically performed ability test for professional German firefighters. The test was conducted as prescribed by German firefighting test regulations (Committee for Firefighting Issues Civil Protection and Civil Defense, 2002). Subjects were tested in a purpose-built practice area, wearing full personal protection gear and a self-containing breathing apparatus (SCBA). The SCBA cylinders were filled with 300 bar (metric unit of the pressure in the SCBA; 1 bar = 100 kPa). The tasks included *ladder climb* (20 m), a 200 m *treadmill walk*, pulling a wire rope *hoist* (15 times) and *crawling* a 50 m *orientation section* in the dark with bottlenecks and a narrow tunnel. Subjects were instructed to perform the SFE safely and as fast as possible but in a pace similar to the work at a real fire emergency scene. The environmental conditions were temperate (20°–30°). HR was measured continuously (Polar, Finland) and ratings of perceived exertion (Borg, 1982) as well as air depletion from the SCBA were taken at the end of the exercise. Total performance time was recorded.

#### Flashover-training (FOT)

The FOT was performed in a special container with a computer-controlled and gas-fired mobile fire simulation training system (Model Firetrainer112, Blaul und Seifert GmbH, Germany). This exercise is also part of periodically performed simulated firefighting exercises in Germany. The training closely simulated the variety and intensity of tasks during a real fire suppression while subjects were exposed to extreme heat (300°) and smoke. Participants were dressed with their personal protective gear (clothing, helmet, gloves, belt, facial mask, boots) and the SCBA for air supply (24.5 kg). Air cylinders were filled with 300 bar. The pressure gauge on the display of the SCBA measured depletion in steps of 10 bar. At the end of the FOT the remaining pressure in the cylinder was read from the SCBA display to determine depletion. The amount of changes in pressure was defined as air depletion. Similar to a real emergency case, each firefighter carried various hand tools (e.g., a hose for fire suppression, a thermal imaging camera). An expert specialized on carrying out flashover trainings supervised the training drill.

Firefighters had to complete the following tasks without interruption:

1. Participants first climbed a 4-m ladder up to the top of the container for access through a door on the top.
2. After opening the door, they had to extinguish the first staircase fire on the stairs that would allow them to enter the container.
3. After successfully extinguishing the staircase fire, firefighters entered the container checking further fire (5 different kinds of fires across the enclosed area (20 m^2^) of the training container: stair case fire again, gas cylinder fire, armchair fire, simulation of an oil fire). Furthermore, every firefighter experienced two unexpected flashovers.
4. The sequence of fires was generated randomly for each subject in order to perpetuate the realistic character of a real fire scene in terms of the unpredictability of a situation at an emergency scene.

Heart rate was measured during the drill, and recovery of HR was measured 1, 3, 5, and 30 min after terminating the drill. Ratings of perceived exertion (Borg, 1982) were recorded after the end of the FOT.

For both, the SFE and FOT, firefighting performance was defined by the TSA-model resulting in a TSA-score as shown in a previous study (Windisch et al., 2017). The TSA-model is a simple formula to quantify the demands of the exercises adding time needed for the exercise, mean heart rate during exercise expressed as percentage of the treadmill determined HR_max_ and air depletion from the SCBA. As the score is based on the function of a z-score, the TSA-score indicates the resultant firefighting performance in relation to the sample mean, with the units measured in standard deviations. A TSA-score of 0 represents the average. We ranked performers according to their TSA-scores into 5 categories based on standard deviations: “Outstanding” (TSA < − 2), “Above Average” (TSA − 1 to − 2), “Average” (TSA − 0.99 to + 0.99), “Below Average” (TSA 1–2), and “Poor” (TSA > 2). Individual performance scores for the TSA should be kept at a minimum achieved through fast completion time, low heart rate as well as low air depletion during the exercise.

#### Strength testing

Strength testing included a standing long jump, legpress 1-RM testing, and maximum handgrip strength. Furthermore, subjects performed maximal possible repetitions of push-ups, partial curl-ups, shoulder press and rowing. The tests were conducted as described in our previously published study (Windisch et al., 2017).

### Data analyses

All data are presented as means ± standard deviation (SD). Data were analyzed using SPSS (Version 23.0, SPSS Inc. Chicago, IL, USA). Data were assumed to be normally distributed if the Shapiro-Wilk's test revealed *p* > 0.05. As all data was normally distributed, parametric tests were carried out. The alpha level was set to 0.05. Paired *t*-tests were calculated to show up differences between different variables of the two firefighting exercises SFE and FOT. Pearson correlations were computed for specific physiological parameters. Statistical significance was set at *p* < 0.05 and correlations were interpreted according to Cohen (1988). Values from 0.10 to 0.29 were considered “small,” 0.30–0.49 “moderate” and ≥0.50 “strong.” In order to identify significant differences in heart rate trajectories of the SFE vs. the FOT, methods of one-dimensional (1D) statistical parametric mapping (SPM) were used (Friston et al., 1995). SPM 1D-analyses were processed as described in Pataky (2010). All data were implemented in Matlab R2016a (8.3.0.532) and normalized to the participants' individual completion time using matlab “interpft” function. Basically, a FFT method was used, where the original vector was transformed to the Fourier domain and then transformed back with desired data points (here 100, representing 100% of completion time). In addition, data was processed with the open source SPM code by Pataky (2016) for two-tailed paired *t*-tests. The critical test statistic threshold that retained a family-wise error rate of α = 0.05 was calculated as described by Pataky et al. (2015). If the SPM{t} trajectory crossed the critical threshold at any time node, the null hypothesis was rejected meaning that the HR trajectories of SFE and FOT are significantly different.

## Results

### Treadmill and strength testing

Total time to exhaustion during treadmill testing averaged 10.3 ± 0.9 min and subjects reached a mean HR_max_ of 182.4 ± 11.9 bpm on the treadmill and a mean absolute VO_2peak_ of 3.59 ± 0.43 l O_2_/min. Corrected for body mass, relative VO_2peak_ averaged 44.1 ± 5.8 ml/min/kg. V_E_ at VO_2peak_ was 124.7 ± 23.3 l/min. VT1 averaged at 2.12 ± 0.27 l/min (59.4% VO_2peak_), RCP averaged at 3.27 ± 0.40 l/min (87.9% VO_2peak_). Expressed as a percentage of HR_max_, VT1 showed up at 70.3 ± 7.7% HR_max_ and RCP at 91.1 ± 6.0% HR_max_.

Subjects performed 115.1 ± 22.6 kg at the legpress, 81 ± 36 partial-curl ups and 25 ± 12 push-ups. Hand grip strength averaged 56.5 ± 8.6 kg and standing long jump 211 ± 22 cm. Mean repetitions to fatigue of shoulderpress and rowing were 22 ± 6 and 10 ± 4, respectively.

### Physiological responses to SFE and FOT

**Figure 1:**
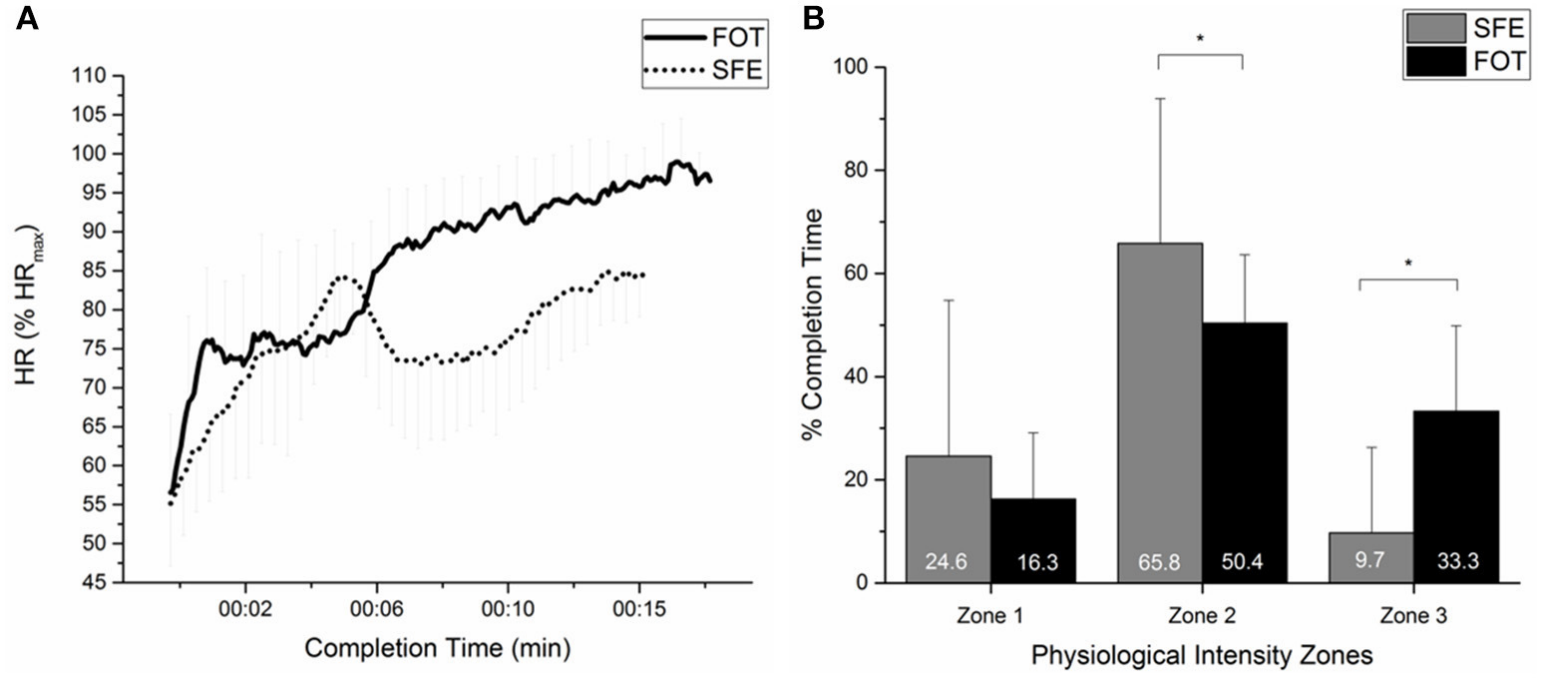
Physiological responses and energy contributions of both exercises **(A)** Kinetics of HR during SFE and FOT, taken as the average value of % of HR_max_ (± SD) of all 16 subjects **(B)** Percentage of time spent in three physiological intensity zones (zone 1 – Z1: below treadmill-determined ventilatory threshold 1 (VT1); zone 2 – Z2: between VT1 and RCP; zone 3- Z3: above treadmill-determined respiratory compensation point (RCP). ^*^Significant difference between physiological intensity zones (*P* < 0.05).

No significant difference (*p* = 0.899) could be found between TSA-scores of the SFE (0.07 ± 2.01) and FOT (0.00 ± 2.12). We could not identify a significant correlation between the two TSA-scores of both exercises (*r* = 0.495, *p* = 0.051). We also analyzed every single component of the TSA-model (completion time, strain in terms of heart rates and air depletion from SCBA). There was a significant difference in completion time between both exercises (*p* = 0.003). Mean completion time of the SFE was 13.2 ± 1.8 min, for the FOT completion time averaged at 15.5 ± 1.2 min. Air depletion from the SCBA were significantly higher (*p* = 0.001) during the SFE with 162 ± 24 bar (54.0 ± 0.08% of full SCBA) compared to the FOT 140 ± 24 bar (46.7 ± 0.08%). Mean HR was lower (*p* = 0.005) during SFE (145 ± 12 bpm) than during the FOT (155 ± 11 bpm). When mean HR was given as a percentage of maximal HR, SFE averaged at 79.9 ± 6.9% HR_max_ and FOT at 85.4 ± 5.2% HR_max_. Figure 1 displays mean HR-kinetics of all subjects across both exercises and the time they spent in the three defined physiological intensity zones (zone 1, zone 2, zone 3).

**Figure 2:**
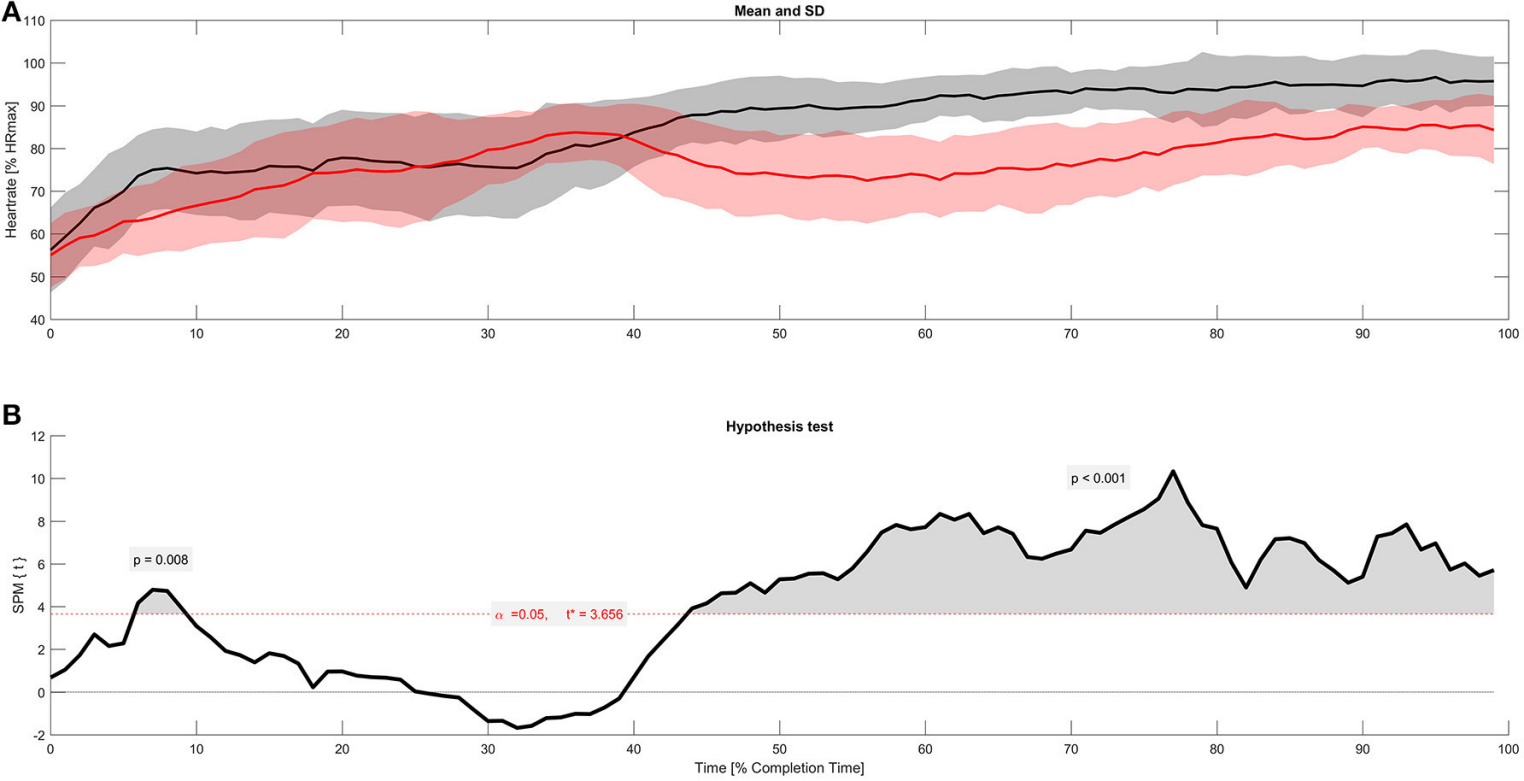
1D-SPM analyses **(A)** Kinetics of HR of all 16 subjects during SFE and FOT, taken as the average value of % of HR_max_ (± SD) and normalized on subject's individual completion time **(B)** 1D-SPM trajectory resulting from 1D-SPM statistics retaining a family-wise error rate of α = 0.05. The critical threshold was at *t* = 3.656.

1D-SPM analyses showed that HR trajectories of SFE and FOT were significantly different over long periods of time (see Figure 2). The critical threshold of 3.656 was first exceeded within the first 10% of completion time but only for 25 s. However, the second time the threshold was exceeded after 43% of mean completion time. From this point the significant difference showed up until the end of the exercise. The precise probability that a supra-threshold cluster of this size – as described by Pataky et al. (2015)—would be observed in repeated random samplings was *p* = 0.008 for the first and *p* < 0.001 for the second cluster.

**Figure 3:**
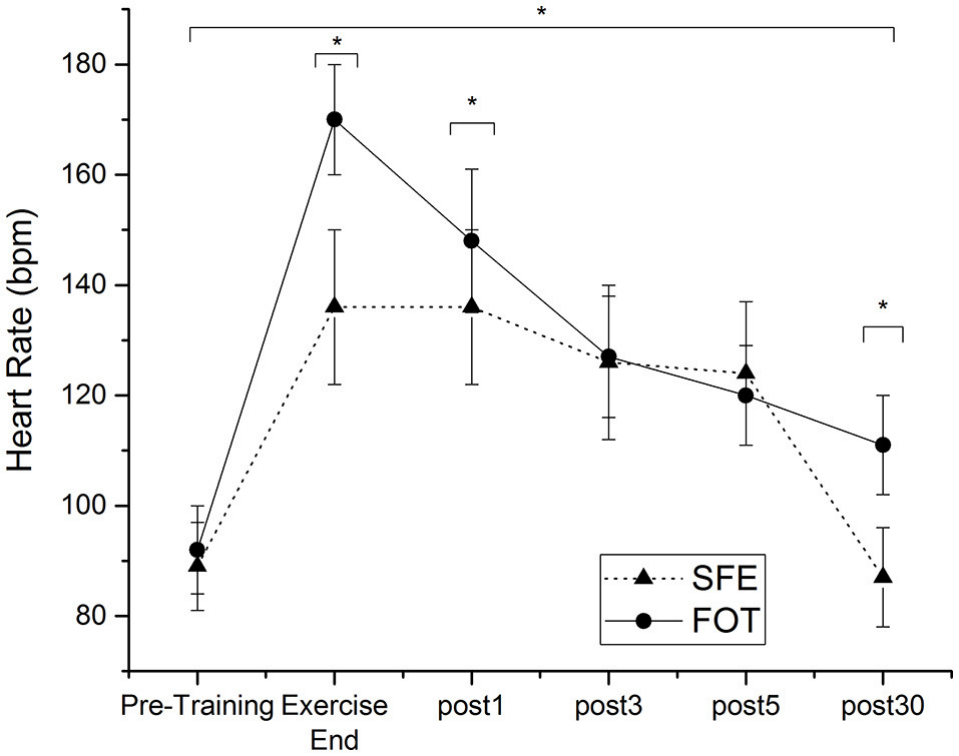
Heart rate (HR) at the end of the exercise and HR recovery 1, 3, 5, and 30 min after SFE and FOT. ^*^Significant difference between heart rate recovery of SFE and FOT (*P* < 0.05).

Heart rates decreased to baseline values after the SFE whereas HR remained elevated compared to pre-training values 30 min after FOT (plus 20 ± 10 bpm). BORG ratings of perceived exertion showed significant differences (*p* = 0.000) between SFE (12 ± 2) and FOT (15 ± 1). Figure 3 gives an overview of HR recovery 1, 3, 5, and 30 min after SFE and FOT.

### Firefighting performance related to fitness measurements

Firefighting performance in terms of TSA-scores during the SFE was strongly correlated to the time subjects spent in zone 1 during this exercise (*r* = −0.547). TSA-scores of the FOT were strongly correlated to the time subjects spent in zone 3 during FOT (*r* = 0.587). Correlations between TSA-scores and its single components completion time, heart rates and air depletion during both exercises are shown in Table 1.

**Table 1:** Correlation matrix between TSA scores, completion time, heart rates and air depletion rates of the standard simulated firefighting exercise (SFE) and the flashover training (FOT) and the time spent in the three defined physiological intensity zones (Zone 1, Zone 2, Zone 3).

**Respective SFE and FOT parameter**	**Respective TSA Score**	
	**SFE**	**FOT**
Completion time	0.611^*^	0.510^**^
HR (% HR_max_)	0.663^**^	0.810^**^
Air Depletion	0.937^**^	0.801^**^
SFE Zone 1	−0.547^*^	−0.344
SFE Zone 2	0.477	−0.403
SFE Zone3	0.184	0.587^*^

* Significant at p ≤ 0.05;

** *Significant at p ≤ 0.01*.

No significant correlations between recovery heart rates of SFE and TSA-Score could be found. For FOT, TSA-score (*r* = −0.760) and air depletion (*r* = −0.904) were highly related to recovery heart rate after 1 min.

TSA-scores were strongly correlated to relative VO_2peak_ during both SFE (*r* = −0.505) and FOT (*r* = −0.621). Significant correlations between TSA-scores of the SFE and strength are shown in Table 2. No significant correlations could be found between TSA-scores of the FOT and strength parameters.

**Table 2:** Correlation matrix between TSA scores, completion time, heart rates and air depletion rates of SFE and FOT and endurance (treadmill testing) and strength (strength testing) characteristics.

	**TSA-score SFE**	**TSA-score FOT**	**SFE completion time**	**FOT completion time**	**SFE HR (%HR_max_)**	**FOT HR (%HR_max_)**	**SFE air depletion**	**FOT air depletion**
VO_2peak_ relative	−0.505^*^	−0.621^*^	−0.074	−0.065	−0.593^*^	−0.693^**^	−0.391	−0.560^*^
VO_2peak_ absolute	−0.144	−0.400	−0.029	−0.140	−0.178	−0.515^*^	−0.091	−0.195
Treadmill time to exhaustion	−0.409	−0.503^*^	−0.094	−0.150	−0.481	−0.482	−0.286	−0.434
Legpress	−0.449	−0.450	−0.259	−0.329	−0.271	−0.314	−0.464	−0.312
Handgrip	−0.188	−0.134	0.133	0.016	−0.380	−0.234	−0.106	−0.060
Curl-ups	−0.085	−0.201	0.007	0.153	−0.144	−0.240	−0.040	−0.338
Push-ups	−0.491^*^	−0.444	−0.211	−0.267	−0.394	−0.293	−0.469	−0.380
Shoulder press	−0.490	−0.198	−0.520^*^	0.008	−0.128	−0.223	−0.479	−0.190
Rowing	−0.343	−0.358	−0.124	−0.213	−0.285	−0.241	−0.343	−0.306
Standing longjump	−0.261	−0.444	0.237	−0.024	−0.542^*^	−0.491	−0.186	−0.424

* Significant at p ≤ 0.05;

** *Significant at p ≤ 0.01*.

Two physiological intensity zones (zone 1, zone 2) were strongly related (*r* > 0.50) to different physiological and strength variables for the SFE, but we found no significant correlation between fitness variables and zone 3 (see Table 3). Only one significant relationship (curl-ups, *r* = 0.720) could be found between the time spent in zone 1 during FOT and strength. Time spent in zones 2 and 3 were strongly (*r* > 0.50) related to many of the physiological and strength variables (see Table 3).

**Table 3:** Correlation matrix between time spent in the three defined physiological intensity zones (Z1, Z2, Z3) for SFE and FOT and aerobic (treadmill testing) and strength variables.

	**SFE Z1**	**SFE Z2**	**SFE Z3**	**FOT Z1**	**FOT Z2**	**FOT Z3**
VO_2peak_ relative	0.603^*^	−0.554	−0.155	0.451	0.556^*^	−0.792^**^
VO_2peak_ absolute	0.181	−0.85	−0.184	0.119	0.672^**^	−0.628^**^
Time to exhaustion during TT	0.566^*^	0.627^**^	−0.033	0.427	0.174	−0.469
VE	0.144	−0.122	−0.054	0.070	0.503^*^	−0.455
VT1	0.351	−0.286	−0.152	0.453	0.143	−0.464
% VO_2peak_ at VT1	0.208	−0.264	0.070	0.409	−0.482	0.067
RCP	0.095	0.017	−0.202	0.062	0.688^**^	−0.596^*^
% VO_2peak_ at RCP	−0.032	−0.040	0.126	−0.038	0.080	−0.034
Legpress	0.210	−0.180	−0.075	−0.325	0.573^*^	−0.205
Hand grip	0.059	0.216	−0.468	−0.394	0.585^*^	−0.161
Curl-ups	0.375	−0.452	0.088	0.720^**^	−0.228	−0.375
Push-ups	0.547^*^	−0.613^*^	0.045	0.210	0.111	−0.252
Shoulder press	0.224	−0.269	−0.051	−0.179	0.403	−0.183
Rowing	0.545^*^	−0.375	−0.352	0.443	−0.019	−0.328
Standing longjump	0.441	−0.382	−0.151	0.409	0.400	−0.634^**^

* Significant at p ≤ 0.05;

** *Significant at p ≤ 0.01*.

## Discussion

### Physiological responses to SFE and FOT

**Figure 4:**
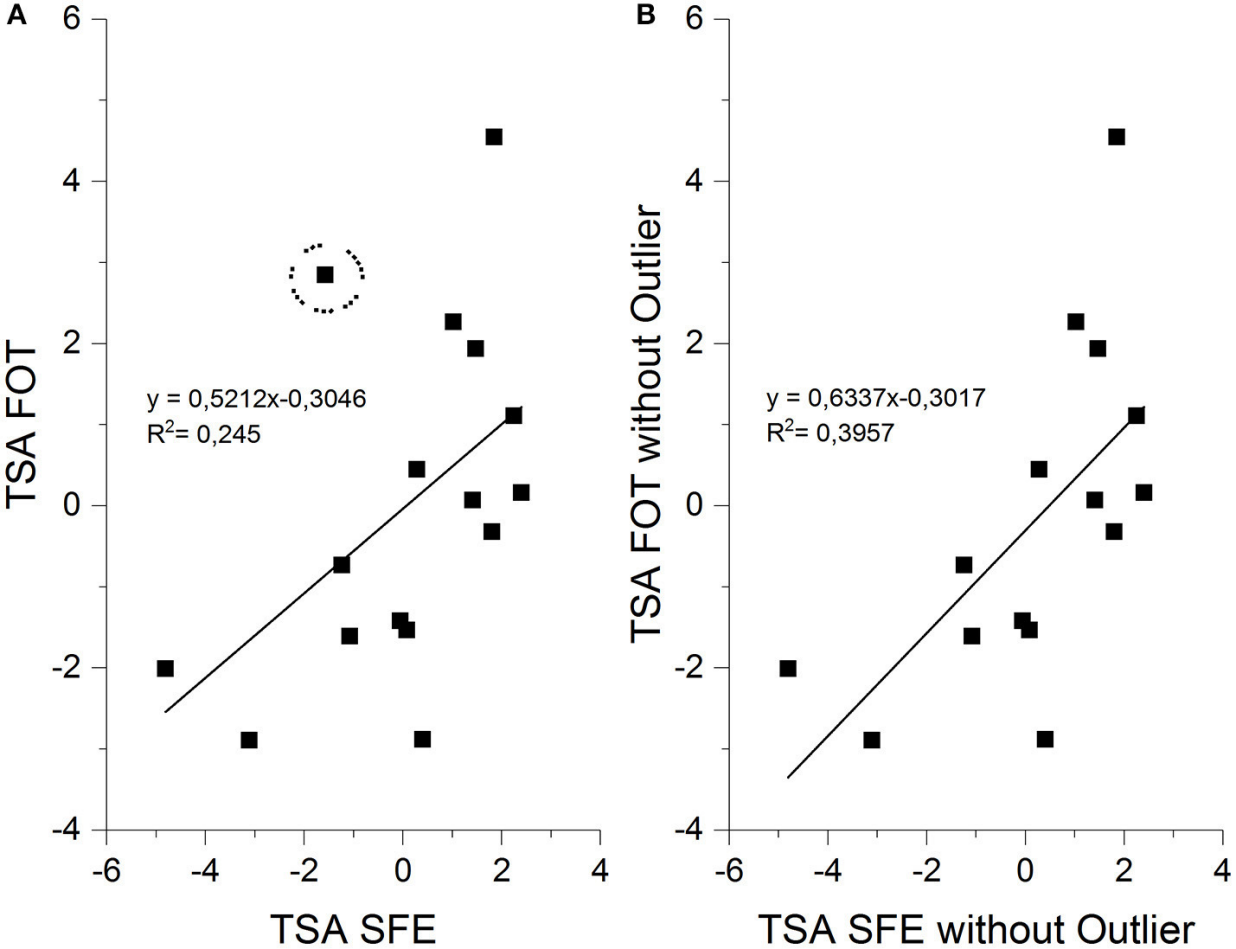
**(A)** Correlation between TSA-scores of both firefighting exercises including all 16 subjects. The outlier was marked by the dotted circle **(B)** Correlation between TSA-scores of both firefighting exercises excluding the outlier that influenced the correlation significantly.

Based on a previous study we considered three parameters important for firefighting performance: time to exercise completion, heart rate during the exercise and air depletion, which we combined in the TSA-model (Windisch et al., 2017). According to this model, the performance of firefighters during the two firefighting exercises in this present study were classified as average. We also investigated the relationship between TSA-scores of both exercises but they were not significantly related to each other. We determined a *p*-value of *p* = 0.051 meaning that significance was narrowly missed. On closer inspection, we found one outlier indicating above average performance (TSA-score: −1.58) in the firefighting exercise without heat compared to below average performance (TSA-score: 2.85) during the firefighting exercise in extreme temperatures. This firefighter showed the largest change of performance as measured by the TSA-score between both exercises. When excluding this outlier from our data, we found a strong and significant relationship between the two TSA-scores of both exercises (*r* = 0.629, *p* = 0.012) (Figure 4). This means that firefighters with a good TSA-score in the standard firefighting exercise showed also a good score in the flashover training. Basically, this strong relationship proves that determining firefighting performance with the TSA-score works and that the TSA-score can be applied to different firefighting exercises.

Regarding the single components of the TSA-model, we found completion time to be shorter during the standard firefighting exercise while participants depleted more air compared to the firefighting exercise in extreme temperatures. One possible reason for these different breathing responses could be hyperthermia-induced hyperventilation meaning that hyperventilation patterns during exercise are changed due to hyperthermia. As shown by Fujii et al. (2008), ventilatory sensitivity to increasing core temperature above the threshold for hyperventilation was lower during moderate exercise in the heat than at rest. This means that ventilation can be attenuated at certain submaximal exercise levels due to hyperthermic conditions in the body (Beaudin et al., 2009; Tsuji et al., 2012).

The physical demands of the standard firefighting exercise in terms of heart rates were considerably less compared to the training in extreme temperatures with smoke and unexpected flashovers. Here our results were in line with the findings of Larsen et al. (2015) comparing also simulated firefighting in very hot and temperate conditions. Mean heart rate as the most commonly reported physiological response to exercises similar to the SFE averaged at 80% HR_max_ with a range from 60 to 90% HR_max_ (e.g., Romet and Frim, 1987; von Heimburg et al., 2006; Del Sal et al., 2009; Williams-Bell et al., 2009; Perroni et al., 2010). The mean HR in our study was in good accordance with the reported values (SFE: 79.0% HR_*max*;_FOT: 85.4% HR_max_). According to these previous studies, the level of physiological strain during firefighting varies depending on the intensity, duration of the physical tasks and environmental stressors. The environmental conditions (high ambient temperature and radiant heat) during the flashover training may have an impact on the physiological strain during the flashover training. It is difficult to establish whether the physiological strain was the result of the physical demands of the activities or the heat stress imposed by the environment, or a combination of both. In this regard, future measurements of core temperature would provide more insights what type of stress impacts heart rate responses. Barr et al. (2010) reported that firefighters were under greater physiological strain in terms of higher heart rates during hot conditions (>40°) compared to temperate conditions (15°–40°) like during the standard simulated firefighting exercise. Similarly, Walker et al. (2015) showed that the completion of a standard work protocol in very hot conditions (up to 100°) resulted in increased core temperature and heart rates (from 74 to 90% HR_max_). Extreme conditions (up to 300°) refer to those encountered during a flashover (Barr et al., 2010). Within this context, it might be that heavier firefighters have more heat capacity and therefore lower core temperature, which can result in lower heart rates. We calculated the relationships between body mass and heart rate but we did not find statistical evidence for a correlation of heat capacity and body weight of subjects in our study (% HR_max_ FOT vs. body mass *r* = 0.485 with *p* = 0.057). However, as statistical significance was just narrowly missed, we think that there can be effects of body mass on the heat capacity of subjects and this aspect should be considered in future studies in combination with core temperature.

The time spent in the three physiological time zones differed significantly between both exercises. During SFE subjects worked for longer time in zone 1 compared to FOT (24.6 and 16.3% of completion time for SFE and FOT, respectively). Zone 1 represents the time subjects worked below ventilatory threshold 1 indicating a high percentage of aerobic metabolism. The SFE revealed significantly lower mean HR with punctual highs and lows, however, never exceeding 86.0% HR_max_. In contrast, the demands of the FOT in extreme temperatures involved continuously increasing heart rates which rarely stayed below VT1. Firefighters spent most of the time in zone 2 (SFE: 65.8%; FOT: 50.4%). Zone 2 represents the time between VT1 and RCP indicating mostly aerobic-anaerobic metabolism. In contrast to SFE (9.7%), during FOT subjects spent one third (33.3%) of the completion time in zone 3 indicating HR above 90% HR_max_. Further, after crossing the threshold to zone 3, heart rate was continuously increasing until to the end of the exercise. Accordingly, heart rate was above firefighter's RCPs indicating that energy production could be heavily supported by anaerobic processes from the middle of the 8th minute until the end of the FOT. During the final 4 min of the FOT firefighters worked on average above 95% of HR_max_, which is considered as very hard physical activity by the American College of Sports Medicine (2013).

The FOT highly challenged subjects, even 30 min post-FOT HR-levels remained elevated compared to pre-exercise. Here, our results go in line with the findings of Smith et al. (1997) and Perroni et al. (2010) who found elevated recovery HR 10 and 30 min post-exercise compared to baseline values. Physiological measures indicated that firefighters experienced symptoms and changes to their health consistent with an overtraining type condition the longer they were exposed to heat (Watt et al., 2016). These changes to health can be enhanced the higher the physiological strain and the longer the subsequent period needed for recovery was (Watt et al., 2016). The increasing core temperature might then also limit firefighting performance. This underlines the need for a high fitness level in order to reduce this strain.

### Firefighting performance related to fitness measurements

Both simulated firefighting exercises of the present study are state of the art exercises to simulate firefighting. Accordingly, both trainings claim to represents job requirements of firefighters. We were interested in how the relationship between firefighting performance in terms of the TSA-score and various fitness parameters changes when the demands of the exercise change from a standard simulated exercise to a simulated exercise that is much closer to a real live scenario in an emergency. The exploration of important relationships was similar to previous work on the topic (e.g., Williford et al., 1999; Rhea et al., 2004; Michaelides et al., 2011). A changing sensitivity of performed tests to assess fitness variables for both exercises would then mean that firefighters need different fitness prerequisites to perform the different exercises successfully. As a result, these changing variables should be considered when conducting fitness tests for endurance and strength to focus on relevant parameters.

Concerning the comparability of the two firefighting exercises, we found a substantial similarity between both exercises although they were different in their demands: a high relative VO_2peak_ can be assumed to be the most important fitness prerequisite for good firefighting performance in both scenarios. Furthermore, the ability to work in specific physiological intensity zones were the second most important aspect of firefighting performance. However, here we found notable differences between the demands of the exercises as zone 1 was highly related to SFE and zone 3 to FOT. To find the most important (VO_2peak_) as well as the changing fitness variables due to the demands of the exercise was the second aim of our study. To know these variables is important to design appropriate exercise programs for firefighters. The present results show that the TSA-model can be applied for firefighting in extreme conditions (heat, smoke, poor visibility, flashovers) as the same kind of fitness parameters (i.e., endurance) were sensitive to predict performance.

These results are in line with our previous research (Windisch et al., 2017), where we found three variables highly related to the TSA-score: VO_2peak_, the time spent in zone 1 during a simulated standard firefighting exercise and breathing frequency. Firefighters with a higher relative VO_2peak_—level and a greater fraction of time spent in physiological intensity zone 1 showed a lower, i.e., better TSA-score. Accordingly, lower TSA-score means that a firefighter is able to operate faster, with less air depletion from the SCBA and lower physical strain indicated by heart rate during the exercise. Unfortunately, due to radiant heat and extreme temperatures, we were not able to collect any data for breathing frequency—the third performance predicting parameter of the TSA-model (Windisch et al., 2017)—during the flashover training.

**Figure 5:**
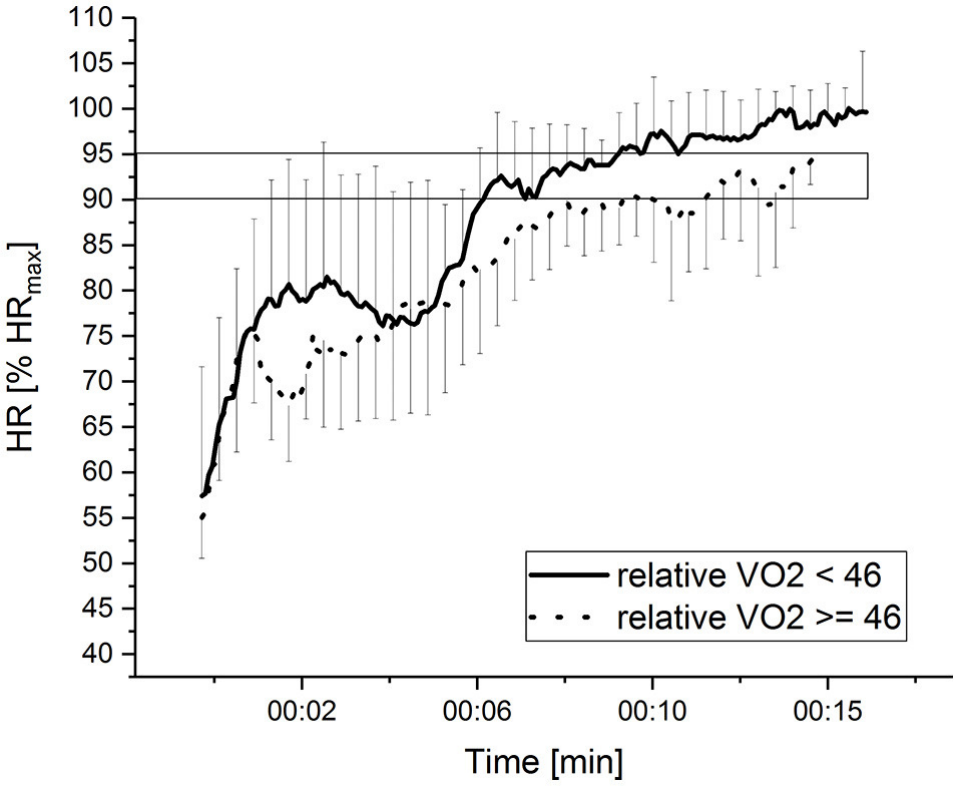
Kinetics of HR during FOT, taken as the average value of % of HR_max_ (± SD) of subjects with a relative VO2peak of ≥ than 46 and < than 46.

Firefighters with a higher relative VO_2peak_ showed a better TSA-score during the flashover training in this study (*r* = −0.621). As relative VO_2peak_ is associated with overall endurance (Jones and Carter, 2000), a high endurance level can be associated to good TSA scores during firefighting in extreme temperatures. The present findings have particular relevance for the published relative VO_2peak_ recommendations for firefighters that vary between 39 and 45 ml/min/kg (O'Connell et al., 1986; Gledhill and Jamnik, 1992; Siddall et al., 2016). These studies recommended minimum values based on firefighting exercises without heat and additional stressors such as flashovers. In general, subjects with a VO_2peak_ < 45 ml/min/kg are classified as “healthy, but sedentary and not so active individuals” while subjects with a VO_2peak_ > 45 ml/min/kg are seen as “recreationally actives” (Laursen and Jenkins, 2002). In our previous study (Windisch et al., 2017), we suggested a minimum VO_2peak_ of 46 ml/min/kg as this was the mean VO_2_-level of the average performers. Dividing now our sample of the present study into two groups (VO_2peak_ < and ≥ 46 ml/min/kg), significantly less physical strain (*p* = 0.002) was found in firefighters with a VO_2peak_ > 46 ml/min/kg. They worked with an average of 91.5% HR_max_ from the 8th minute until the end of FOT compared to subjects with a VO_2peak_ of less than 46 ml/min/kg who worked with an average of 97.5% HR_max_ (see Figure 5). Arguments for this minimum VO_2peak_ were also underlined by Périard et al. (2011) who found that in environments with high temperatures, exhaustion occurred after crossing 96% of HR_max_ and was accompanied by significant declines in stroke volume (15–26%), cardiac output (5–10%), and an increase in mean arterial pressure (9–13%). A minimum VO_2peak_ of 46 ml/min/kg in combination with good anaerobic metabolism therefore could help to reduce possible negative health consequences for firefighters as it would allow them to keep strain below 96% HR_max_. However, this recommended minimum value needs to be investigated in future studies to prove that it is a justified threshold.

Endurance training cannot only help to increase VO_2peak_ but also to accelerate HR recovery, which was strongly related to VO_2peak_ in our study (*r* > 0.613). This goes in line with other studies observing faster HR recovery in subjects with higher VO_2peak_ (Darr et al., 1988; Du et al., 2005). Faster HR recovery will then also have an indirect effect on a better TSA-score because we found that air depletion rates from the SCBA were strongly related to HR recovery 1, 3, and 5 min post-FOT. However, it should also be considered that endurance training can improve the sweating response, which accelerates dehydration. Given the fact that sweat can hardly evaporate inside the protective clothing, the increased sweat accumulation could add some discomfort for the firefighter (Aoyagi et al., 1998). Greater sweating makes it difficult to maintain body core temperature at lower level. However, this does not mean that firefighters should not keep a high endurance level. Cross-sectional comparisons between groups of high and low aerobic fitness have revealed that a high aerobic fitness is associated with extended tolerance time when protective clothing is worn (McLellan, 2001). According to McLellan (2001), elevations in core temperature that occur with long-term training in normal training sessions may familiarize the more fit subjects to the discomforts of exercise in the heat.

#### Study limitations

Measuring core temperature would provide more insights into what type of stress—physical demand or heat stress—impacted more on heart rate responses. Together with the measurement of dehydration this would be an important aspect to be considered in future studies because there was no data available from the present study (e.g., measurements of mass prior to and following the exercises). Furthermore, the mental stress-induced tachycardia due to flashovers could also influence heart rate responses. Unfortunately, we were not able to separate HR responses exactly between the different fires as the sequence of fires was generated randomly for each subject in order to perpetuate the realistic character of a real fire scene in terms of the unpredictability of a situation at an emergency scene. The fires were overlapping during the exercises which made it impossible to attribute mental stress-induced heart rate responses to flashovers. Finally, our subjects were instructed to complete the different exercises as fast as possible but in a pace similar to the work at a real fire emergency scene. Mean firefighting service time of our subjects was 17 years, so we expected them to make an appropriate assessment about the pace they worked with our instruction to complete the exercises as fast as possible. However, a real emergency (in terms of putting out a real fire) might change the judgment about the pace a firefighter can fight the fire.

## Conclusions

Firefighting performance can be determined by the TSA-model adding time for exercise completion, physical strain indicated by mean heart rate and air depletion from the SCBA. The comparison of the two investigated firefighting exercises showed that the fitness contribution differs with respect to the different demands of the exercise. For standard firefighting exercises like the exercise under temperate conditions in our study, it is important that firefighters are able to spend a great portion of time below VT1. For simulated firefighting in extreme temperatures with smoke, poor visibility and unexpected flashovers, firefighters need a good fitness level in order to spend as little time as possible in zone 3. From all variables researched in our study, we found relative VO_2peak_ to be the primary physiological variable related to the different aspects of firefighting, strengthening our plea to consider endurance as the most important prerequisite for firefighting. For practical application, we strongly recommend firefighters to sustain a high level of relative VO_2peak_, VT1 and RCP. We measured energy contributions during simulated firefighting very indirectly in this study by relating HR during the firefighting exercises to the established HR-levels at VT1 and RCP during the maximum treadmill test, this is also an indirect indicator for the different metabolisms that are important for successful firefighting. Here the data can help to design appropriate exercise programs for firefighters. Furthermore, we recommend to work on recovery heart rates with endurance training. These parameters can easily be tested in the laboratory, which allows for valid standardization and requires fewer resources compared to simulated firefighting exercises. We recommend a minimum VO_2peak_ of 46 ml/min/kg and regular standardized endurance tests, to allow firefighters to perform their jobs healthy and safely.

## Ethic statements

Full written and verbal details about the study were provided and all subjects gave written informed consent before participating in the study. The ethic statement for this study was approved by the Dean of the Faculty of Sports and Health Sciences of the Technical University of Munich. All procedures performed in this study involving human participants were in accordance with the ethical standards of the institutional and/or national research committee and with the 1964 Helsinki declaration and its later amendments or comparable ethical standards.

## Author contributions

Study conception and design by SW, AS, DH; Data acquisition by SW. Data analysis and/or interpretation by SW, WS, DH; Drafting of the manuscript by SW; Revising by WS, DH; Final approval of manuscript provided by SW, WS, DH, AS.

### Conflict of interest statement

The authors declare that the research was conducted in the absence of any commercial or financial relationships that could be construed as a potential conflict of interest.

## Abbreviations

BMI: Body-Mass-Index
FOT: Flashover training
HR: Heart rate
HR_max_: Maximum heart rate
RCP: Respiratory compensation point
RER: Respiratory exchange ratio
SCBA: Self-containing breathing apparatus
SD: Standard deviation
SFE: Standard simulated firefighting exercise
TSA: Time–strain-air depletion
VCO_2_: Carbon dioxide output
V_E_: Ventilation
VO_2_: Oxygen consumption
VO_2peak_: Peak oxygen uptake
VT1: Ventilatory threshold 1.
